# An experimental study on the influence of low-contrast optotypes on the performance of multi-toric lenses for astigmatism compensation

**DOI:** 10.1007/s44402-026-00025-3

**Published:** 2026-02-27

**Authors:** Diana Gargallo, Anabel Martinez-Espert, Laura Remón, Jorge Ares

**Affiliations:** 1 Applied Physics, University of Zaragoza, Zaragoza, Spain; 2 Departamento de Optica y Optometria y Ciencias de la Vision, Universitat de Valencia, Burjassot, Spain

**Keywords:** Astigmatism, Low-contrast visual acuity, Multi-toric lenses, Rotational stability

## Abstract

**Purpose:**

Achieving good visual quality with a conventional spherocylindrical (CSC) lens requires precise alignment with the principal meridians of the astigmatism to be corrected. Despite advancements in stabilisation, current contact lens and intraocular lens designs face limitations on this regard. This study introduces different trizonal multi-toric (TMT) lens designs endowed with three concentric zones to provide 3.50 D of astigmatic correction, with increased tolerance to rotational errors. Experimental results using low-contrast optotypes are also presented for the best purposed design.

**Methods:**

First, several variants of 3.50 D astigmatic TMT designs were created and evaluated by means of retinal image simulation. The optical TMT design was then selected based on its Visual Strehl Ratio (VSR) performance and simulated image quality. Subsequently, a double-blind clinical study was conducted using an adaptive optics visual simulator and low-contrast optotypes. The study compared the maximum low-contrast visual acuity (LCVA) achieved with the best TMT design to that of a conventional spherocylindrical (CSC) design under varying degrees of rotational error.

**Results:**

Numerical image simulations confirmed the superior rotational tolerance of the TMT designs, compared with CSC lenses, maintaining acceptable image quality up to ±7.50°, particularly with a 40% central zone. Clinical evaluation showed that the 3.50 D astigmatic CSC design provided better LCVA than the best TMT design when properly aligned; however, this advantage decreased quickly with increasing rotational error. At ±7.50°, the CSC suffered a nearly two-line loss (+0.19 logMAR) compared with a one-line loss (+0.07 logMAR) achieved for the best TMT design tested here.

**Conclusions:**

Retinal image simulations have shown that TMT designs with a 40% central zone exhibit superior tolerance to rotational errors than CSC lenses. Moreover, the LCVA of the TMT design used in this study remained more stable under rotational errors than that of a CSC lens.

Key Points

- The correction of astigmatism with spherocylindrical lenses is limited significantly by rotational instability, which directly impacts patients’ visual quality.
- Numerical image simulations for 3.50 D of astigmatism confirmed that trizonal multi-toric designs offer superior rotational tolerance compared with conventional spherocylindrical lenses, maintaining acceptable retinal image quality even with rotational errors of up to ±7.50°.
- Clinical evaluation confirmed that while conventional spherocylindrical lenses may provide better low-contrast visual acuity when properly aligned, this advantage significantly decreased with rotation. At ±7.50°, trizonal multi-toric lenses demonstrated superior mean low-contrast visual acuity, with notably less vision loss compared with conventional lenses.

## Introduction

Astigmatism is a common refractive error of the eye, primarily caused by rotationally asymmetric curvatures of the cornea and crystalline lens. This optical imperfection leads to blurred vision at all distances and often coexists with other refractive errors such as myopia or hyperopia. Astigmatism can be classified as regular or irregular, with regular astigmatism being the most prevalent and typically correctable with spectacles, contact lenses (CL) or refractive surgery. Epidemiological studies estimate that astigmatism affects approximately 30% of the population, making it one of the most widespread visual conditions worldwide [1].

The correction of regular astigmatism has traditionally been achieved using toric lenses in various forms. However, due to their operating principle, toric lenses require precise alignment with the principal meridians of the ocular astigmatism (Ast) in order to deliver optimal visual performance. When the axis of the compensating cylinder is misaligned with that of the reference cylinder, residual astigmatism (Ast_res) is induced. Using power-vector formulae [2, 3], it can be readily shown that this residual astigmatism is mixed in nature, with a zero spherical equivalent. Furthermore, its magnitude can be calculated as:

1 $$Ast\_res = Ast \times 2\sin (\alpha \_rot)$$

where *α*_rot denotes the angular misalignment (in degrees) between the principal meridians of the toric compensation and the astigmatic eye.

For practical purposes, a first-order approximation of this expression (commonly known as the ‘3.33% rule’) is widely used in clinical settings due to its computational simplicity [4]. Basically, for each degree of rotation from the intended axis, 3.33% of the intended cylindrical power correction is lost, leading to a proportional increase in residual astigmatism. Considering this relationship, when the rotational misalignment reaches 30°, the Ast_res is equal in magnitude to the original astigmatism intended for correction [4, 5]. The impact of this residual error on visual quality increases with the magnitude of the astigmatism [6]. This behaviour represents a significant limitation in clinical situations where the stability of toric lenses is compromised, such as with CL or intraocular lenses (IOLs).

With this scenario, several stabilisation strategies, such as prism ballast and dynamic stabilisation, have been implemented for toric CLs. Similarly, toric IOLs have seen novel tools to improve alignment during implantation [7]. Despite these developments, these approaches remain limited in efficacy and are not free of drawbacks [8].

To mitigate the degradation of visual quality induced by toric lens rotation, a new trizonal multi-toric (TMT) design has recently been proposed [9]. TMT lenses incorporate multiple concentric zones with distinct astigmatic axis orientations, aiming to provide effective correction across a range of rotational positions. A TMT lens designed to correct 3.50 D of astigmatism has demonstrated improved tolerance to misalignment angles up to ±7.50°, compared with its conventional spherocylindrical (CSC) counterpart.

The working principle of TMT lenses shares some similarities with simultaneous vision multifocal CL used in the correction of presbyopia [10]. While multifocal lenses integrate different spherical powers within the optical zone to improve tolerance to defocus across viewing distances [11], TMT lenses combine multiple astigmatic powers to increase tolerance to rotational errors. However, optical multiplexing within the pupil zone can negatively affect image quality. Studies on multifocal CLs have reported declines in contrast sensitivity [12, 13], along with visual artefacts such as halos [14, 15]. Given this similarity, it is plausible that TMT lenses may also induce similar optical side effects.

In this context, the aim of the present study was to evaluate the limitations of a TMT design under low-contrast conditions, in comparison with a conventional toric lens. To this end, an initial optimisation was carried out to identify the TMT configuration that maximised the Visual Strehl Ratio (VSR) for 3.50 D of astigmatism. In a second stage, the rotational tolerance of the best TMT design was assessed using an adaptive optics visual simulator and low-contrast optotypes in a sample of participants. Moreover, to facilitate the reproducibility of the computational and experimental results by other researchers, a complete description of the TMT phase profile designs is provided.

## Methodology

First, a series of different TMT designs were implemented to compensate a cylindrical refractive error of −3.50 DC × 180°, for a pupil diameter of 4.50 mm. In a second stage, to determine the most suitable TMT design for the intended refractive prescription, the image quality tolerance to rotation errors was evaluated by means of retinal image simulation. Finally, the TMT design that yielded the best results in these tests was subsequently evaluated in a population of real patients using the adaptive optics visual simulator (VAO, Voptica SL,voptica.com/vao).

## Trizonal Multi-toric Elements Design

The optical zone consists of three concentric zones defined into a total aperture radius of ${R}_{3}$ = 2.25 mm. In general terms, the total optical phase of the element $\varPhi \left(x,y\right)$ can be described piecewise for each concentric zone as a second order Zernike polynomial sum as:

2 $$\varPhi \left(x,y\right)=\left\{\begin{array}{c}{a}_{4\left(1\right)}{Z}_{4}\left(\frac{r}{{R}_{3}},{{\rm{\theta }}}\right)+{a}_{5\left(1\right)}{Z}_{5}\left(\frac{r}{{R}_{3}},{{\rm{\theta }}}\right)+{a}_{6\left(1\right)}{Z}_{6}\left(\frac{r}{{R}_{3}},{{\rm{\theta }}}\right)for\,0\le r < {R}_{1}\\ {a}_{4\left(2\right)}{Z}_{4}\left(\frac{r}{{R}_{3}},{{\rm{\theta }}}\right)+{a}_{5\left(2\right)}{Z}_{5}\left(\frac{r}{{R}_{3}},{{\rm{\theta }}}\right)+{a}_{6\left(2\right)}{Z}_{6}\left(\frac{r}{{R}_{3}},{{\rm{\theta }}}\right)for\,{R}_{1}\le r < {R}_{2}\\ {a}_{4\left(3\right)}{Z}_{4}\left(\frac{r}{{R}_{3}},{{\rm{\theta }}}\right)+{a}_{5\left(3\right)}{Z}_{5}\left(\frac{r}{{R}_{3}},{{\rm{\theta }}}\right)+{a}_{6\left(3\right)}{Z}_{6}\left(\frac{r}{{R}_{3}},{{\rm{\theta }}}\right)for\,{R}_{2}\le r < {R}_{3}\end{array}\right.$$

Where $\ {Z}_{{{\rm{j}}}}$ denotes the *j*th Zernike polynomial as defined by the American National Standards Institute standard [16], ${a}_{j(k)}$ is the coefficient associated with the polynomial $\ {Z}_{j}$ in zone *k*, $\frac{r}{{R}_{3}}$ and $\theta$ represents the normalised radial and angular coordinates, respectively, following the same standard definition. Moreover, ${R}_{1}$ and ${R}_{2}$ correspond to the radii of the central zone (*k* = *1*) and the first peripheral zone (*k* = *2*), respectively. Finally, ${a}_{4(k)}$ corresponds to the vertical second-order astigmatism present at the *k*th zone, ${a}_{5(k)}$ corresponds with defocus and ${a}_{6(k)}$ to the oblique second-order astigmatism at the same zone.

The Zernike coefficient values for each *k*-zone were calculated based on the desired spherocylindrical refraction according to the following expression:

$${a}_{4,k}=-\frac{{R}_{3}^{2}}{2\surd 6}\cdot \left(\frac{{C}_{k}}{2}\right)\cdot \cos \left(2{\alpha }_{k}\right)$$

3 $${a}_{5,k}=-\frac{{R}_{3}^{2}}{4\surd 3}\cdot \left({S}_{k}+\frac{{C}_{k}}{2}\right)$$

$${a}_{6,k}=-\frac{{R}_{3}^{2}}{2\surd 6}\cdot \left(\frac{{C}_{k}}{2}\right)\cdot \sin \left(2{\alpha }_{k}\right)$$

where *S*_*k*_ and *C*_*k*_ represent the spherical and cylindrical power of the *k*th zone, respectively, and ${\alpha }_{k}$ is the orientation angle of the cylinder in that same zone.

All TMT designs developed in this work share the same spherocylindrical refraction in the central zone (*k* = 1) so: ${S}_{1}$ = 0.00 D, ${C}_{1}$= −3.50 D and ${\alpha }_{1}$ = 180°, designated as SC_0. That is, the central zone was chosen to be aligned with the nominal astigmatic axis of the prescription. The peripheral rings ($k$= 2 and $k$= 3) also share the same spherical and cylindrical power so that ${S}_{2}$ = ${S}_{3}$ = 0.00 D and ${C}_{2}$ = ${C}_{3}$ = −3.50 D. However, to increase the tolerance to rotation, the cylinder axis orientation ${\alpha }_{k}$ in the peripheral zones was intentionally offset by ±5° from the prescribed axis. The results of these rotations were defined according to the TABO axis reference system [17] as $\alpha$= 5° (SC_5) and *α* = 175° (SC_175).

**Fig. 1:**
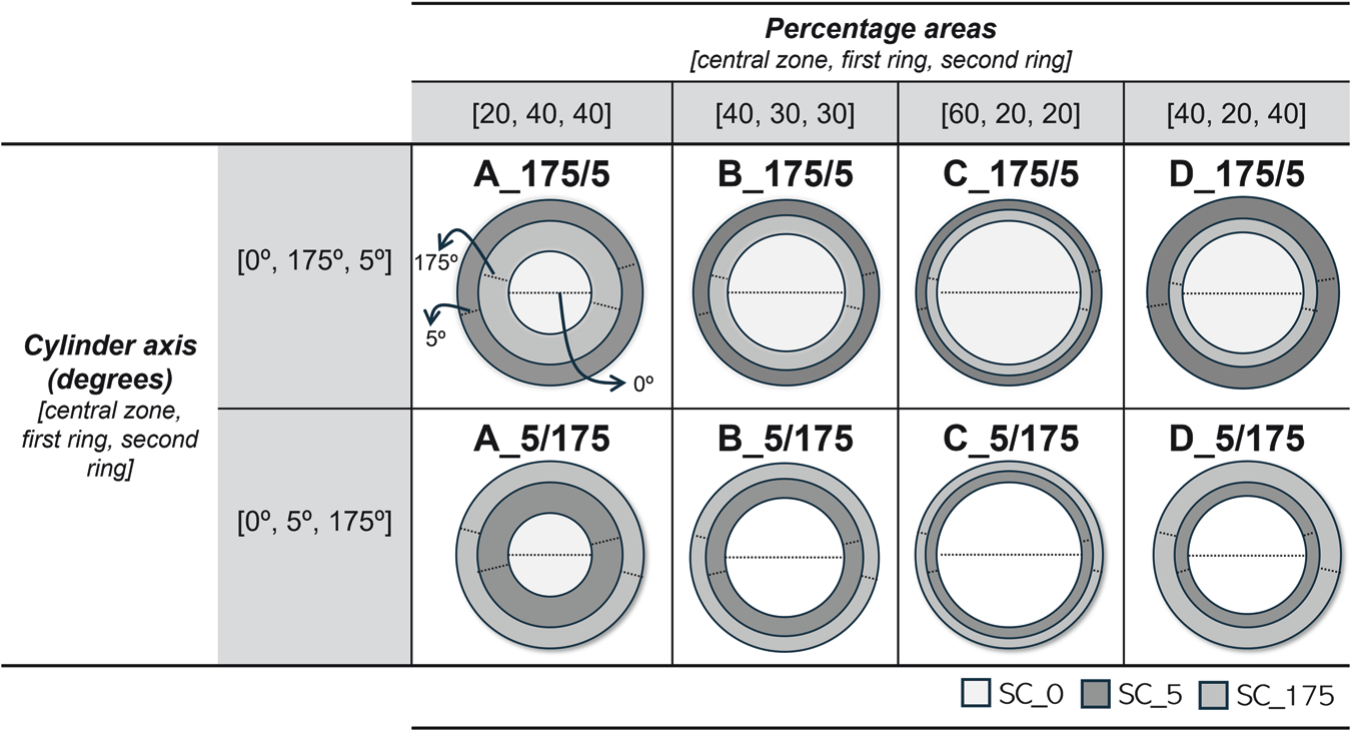
Details of the trizonal multi-toric (TMT) designs. Bracketed values indicate the percentage area of each zone [central zone, first ring and second ring]. The dashed line indicates the direction of the negative cylinder axis. SC_0: Spherocylindrical (SC) correction with the cylinder axis aligned to the astigmatic axis. SC_5 and SC_175: the same spherocylindrical correction, but with the axis oriented at 5° and 175°, respectively, following the TABO convention [17]. The total diameter of the element was 4.50 mm.

A total of eight different designs were evaluated to determine the most suitable configuration for the refractive prescription selected in this work. This evaluation involved exploring various area distribution balances amongst the three zones of the design, as well as alternating the direction of the cylindrical compensation rotations applied to the peripheral zones. Each design was identified by a code consisting of a letter (A, B, C, or D) and two numbers separated by a forward slash. The letter defines the percentage area distribution of the central, first-ring, and second-ring zones (A[20,40,40]:; B[40,30,30]:; C[60,20,20]:; D[40,20,40]:), while the numbers specify the orientation of the astigmatic compensation axes of the first and second rings. Since two orientations were tested, this yielded four design pairs: {A_175/5, A_5/175}, {B_175/5, B_5/175}, {C_175/5, C_5/175}, and {D_175/5, D_5/175}. Figure 1 provides a detailed graphical description of the implemented designs.

## Retinal Image Simulations to Find the Best Potential Design

Retinal image simulations were performed to evaluate the performance of the TMT designs in comparison with CSC compensation under different rotational errors. For the retinal image simulation, a reduced emmetropic eye model was employed with an aperture radius of 2.25 mm, a refractive index of 1.333 and an axial length of 22.22 mm [18]. Following an analogous method to that described by Ares et al. [11], the ametropic and compensating elements were combined into a single thin phase plate placed at the single refracting surface of the model eye, sharing the same aperture as the model itself, which also defined the system aperture. Based on this model, the point-spread function (PSF) [19] was obtained as the intensity of the Fourier transform of the complex pupil function. The simulated retinal images were then generated by convolving this PSF with the paraxial image of a given optotype using MATLAB v.23.2 (mathworks.com).

Simulated retinal images were developed under ideal alignment with the patient’s astigmatic axis as well as with controlled rotational misalignments of 2.50°, 5.00° and 7.50°, applied in both negative (clockwise) and positive (counterclockwise) directions. A high-contrast letter line consisting of the letters ‘S’, ‘P’ and ‘B’ (0.00 logMAR visual acuity (VA)) was used to reveal the effects of image degradation caused by aberrations. It is noteworthy that according to the ‘3.33% rule’, every 2.50° increment in rotational misalignment results in approximately 0.15 D of induced spherical equivalent error. A priori, this relatively small, induced error warrants that the chosen rotational sampling is sufficient to capture the effects of misalignment in the simulations.

Moreover, to provide an objective assessment of image quality, the VSR was calculated for each condition [20]. This parameter has been demonstrated to be an objective and robust metric for quantifying optical quality and predicting visual performance from wavefront aberrations, showing a strong correlation with VA [21, 22].

## Clinical Evaluation

The double-blind clinical study was conducted in accordance with the tenets of the Declaration of Helsinki and was approved by the Ethics Committee for Clinical Research of Aragon (CEICA). Written informed consent was obtained from all participants prior to enrolment.

Inclusion criteria included healthy individuals between 18 and 35 years of age, with a spherical refractive error less than ±4.00 D, an astigmatic error <1.00 D and no history of refractive surgery. CL wearers were instructed to discontinue lens use at least 48 h before measurements.

Sixteen adult subjects were included in this study, with a mean age of 22.75 ± 3.29 years (range: 19–32 years). One eye per participant was analysed, corresponding to the sighting dominant eye, to ensure the greatest possible visual comfort during the trials. In total, 16 eyes were evaluated: 10 were right eyes, and six were left eyes. The mean subjective spherical refractive error was −1.39 ± 1.30  D (range: 0 to −3.50 D) and the mean cylindrical component was −0.20 ± 0.30 D (range: 0 to −0.75 D), expressed in minus cylinder notation. The mean best-corrected distance VA was −0.08 ± 0.05 logMAR (range: −0.18 to +0.00 logMAR), and the mean root mean square of higher-order aberrations was 0.14 ± 0.06 μm (range: 0.08 to 0.26 μm) for a 4.5 mm pupil diameter.

To assess the visual performance of the best TMT found by means of retinal image simulation quantitatively, clinical measurements were conducted using an adaptive optics visual simulator (VAO, Voptica SL, voptica.com/vao) [23]. This commercial device integrates a Hartmann–Shack sensor for wavefront aberration measurement and a liquid crystal on silicon display to simulate custom optical phase profiles.

Clinical assessments were performed by inducing a −3.50 DC × 180° astigmatic ametropia and its corresponding optical compensation. In order to do so, the VAO simultaneously corrected each participant’s refraction and induced this astigmatic error using either the CSC design or the previously described B_175/5 design, under both aligned and misaligned (rotational error) conditions.

Monocular VA was assessed under photopic conditions (80 cd/m²) using a Snellen E optotype displayed on the device’s integrated organic light-emitting diode screen. Each line contained five ‘E’ letters randomly oriented (up, down, right or left), with spacing equal to one letter width. Lines were presented in isolation from +0.40 to −0.20 logMAR in 0.10 logMAR steps. Participants indicated the orientation of each ‘E’. The test was stopped when three or more letters were missed on the same line. Final VA was calculated from the last line read, adding +0.02 logMAR for each letter missed during the test. The tumbling E optotype provided a standardised, orientation-independent measure and enabled applicability of the results in future studies involving children.

Measurements were performed using the CSC and the best TMT designs. For each design, low-contrast visual acuity (LCVA) (30%) was assessed in the aligned position and under controlled rotational misalignments of ±2.50°, ±5.00° and ±7.50°. High-contrast VA (HCVA, 100%) was measured only in the nominal aligned position, which served as the best-case reference (HCVAref). With this scenario, each participant completed 16 VA assessments (eight with the CSC design and eight with the TMT design) in a single 50-min session. All measurements were performed without cycloplegia. Trials were presented in randomised order to minimise bias, and two short breaks were scheduled to reduce fatigue effects. Both the examiners and participants were masked as to the optical condition being tested, preserving the double-blind nature of the study.

All acquisitions were performed using a fixed simulated pupil diameter of 4.50 mm, while the participants’ actual pupil size was monitored throughout the session, with a mean diameter of 6.96 ± 1.04 mm (range: 4.88–8.04 mm).

## Analysis

First, the behaviour of the VSR, along with the appearance of the simulated image, was used to identify the most promising design to be tested in the final clinical study using the VAO device. Next, descriptive statistics were applied to characterise the population data.

Box plots and statistical tests were used to compare the maximum VA achieved by each optical design, as well as their tolerance to rotational errors for low-contrast optotypes. The normality of the data was verified using the Shapiro–Wilk test, which confirmed a normal distribution. Consequently, statistical comparisons were performed using the paired Student’s *t*-test, with a significance level of 0.05 being applied throughout. Potential differences between negative and positive rotation errors were evaluated in advance to determine whether the results could be pooled.

Finally, to evaluate the robustness of visual performance under conditions of low-contrast and rotational misalignment, a dual-axis bar chart was used to represent the VA loss for both the CSC and the best-performing TMT design. Visual quality degradation was quantified relative to the ideal case of perfect alignment and high contrast.

## Results

### CSC and TMT Retinal Image Simulations

**Fig. 2:**
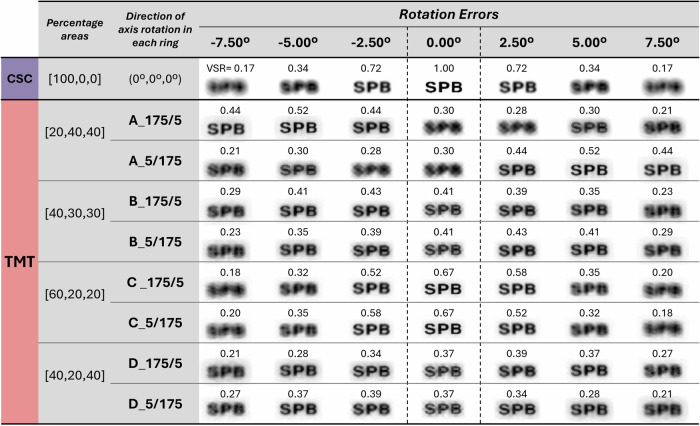
Simulated retinal images of CSC and TMT designs under different conditions. Rotations from −7.50° (clockwise) to +7.50° (counterclockwise) were simulated using an optotype with 0.00 logMAR visual acuity. The upper value of each image corresponds to the VSR. A–D indicate the percentage distribution of the area corresponding to the central zone, the first ring, and the second ring: A[20,40,40], B[40,30,30], C[60,20,20], and D[40,20,20]. CSC conventional sphero-cylindrical, TMT trizonal multi-toric, VSR visual Strehl ratio.

Figure 2 shows simulated retinal images of a 0.00 logMAR VA optotype for both the CSC and TMT designs, evaluated with rotational errors of 0.00°, ±2.50°, ±5.00° and ±7.50° in a model eye with a spherocylindrical refraction of −3.50 DC × 180°.

Under optimal alignment (0.00°), the CSC lens delivered the highest visual quality (VSR = 1.00) as expected. A 2.50° rotation reduced image quality (VSR = 0.72), while a 5.00° rotation caused a steep decline (VSR = 0.34), severely compromising optotype recognition (see first row of Fig. 2). At small rotations (2.50°), the CSC lens deteriorated less than the TMT designs; however, at larger rotations (≥5.00°), TMT designs exhibited greater tolerance.

In the aligned position, configurations with a large central zone (designs C_175/5 and C_5/175) achieved the best performance (VSR = 0.67). However, their visual quality progressively degraded from good to unacceptable as the rotational error increased beyond ±5.00°. In contrast, designs with a smaller central zone (designs A_175/5 and A_5/175) exhibited poor image quality under optimal alignment; nonetheless, their performance improved notably when the lens was rotated toward the orientation of the first concentric ring. Design A_175/5 peaked at a VSR of 0.44 with a −7.50° rotation, while design A_5/175 reached the same value at +7.50°.

Between these extremes, designs B_175/5 and B_5/175, featuring an intermediate central zone, showed acceptable visual quality at 0.00° (VSR = 0.37) and demonstrated enhanced tolerance to rotation errors up to ±7.50°. Specifically, design B_175/5 showed slightly improved optotype image quality with negative rotations, whereas design B_5/175 responded more favourably to positive rotations. Finally, the designs with unequal peripheral ring size (D_175/5 and D_5/175), prioritising the outermost ring and a 40% central area, showed acceptable visual quality without rotation (VSR = 0.37) and considerable tolerance to rotation.

When comparing ring orientations in the 175/5 and 5/175 designs, their rotational behaviour was asymmetric between negative and positive directions. The 175/5 design exhibited greater tolerance to negative rotations, whereas the 5/175 design performed better under positive rotations, consistent with the orientation of its first peripheral ring.

Based on these simulations, the B_175/5 design was chosen as the optimal candidate for clinical evaluation because it demonstrated robust visual performance both when aligned and with rotational errors up to ±7.50°, indicating high stability against misalignment. Although other designs, such as design D, also showed acceptable rotational tolerance, the VSR obtained with design B was generally consistently higher, suggesting a potential better preservation of image quality in low-contrast scenarios.

### Clinical Evaluation

**Fig. 3:**
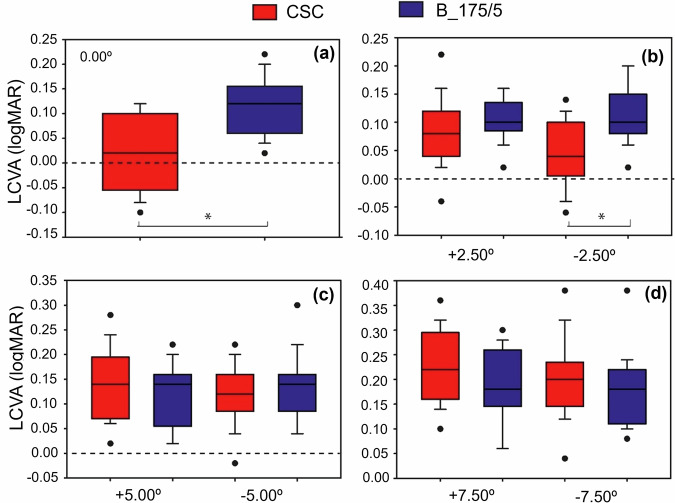
Box plots of low contrast visual acuity (LCVA) for CSC and B_175/5 designs under different rotations. Box plots of low contrast visual acuity (LCVA) for CSC and B_175/5 designs under different rotations **a** on-axis, **b** 2.50°, **c** 5.00° and **d** 7.50°. Each box shows the median (central line), first (Q1) and third (Q3) quartiles (box edges) and the full range (whiskers). * Statistically significant differences (*p* < 0.05). CSC conventional sphero-cylinder, B_175/5 Optimal TMT (trizonal multi-toric) design with cylinder axes at 175° (first ring) and 5° (second), and area distribution of [40, 30, 30]%.

Figure 3 shows box plots of LCVA for all samples evaluated with the CSC and B_175/5 designs under different rotational conditions: 0.00°(a), ±2.50°(b), ±5.00°(c) and ±7.50°(d). In the aligned condition (0.00°), the CSC design provided significantly better LCVA than B_175/5 (*p* < 0.01). This difference remained significant at ±2.50°, although only for the negative rotation. At ±5.00°, LCVA values were similar between CSC and B_175/5 and at different orientations, with no statistically significant differences. At ±7.50°, mean LCVA values were around 0.18 logMAR for B_175/5 and between 0.20 and 0.23 logMAR for CSC, with no statistically significant differences between designs.

Statistical analysis showed no significant differences between positive and negative rotations for either design. Specifically, *p*-values for CSC were 0.07 (±2.50°), 0.40 (±5.00°) and 0.20 (±7.50°), while for B_175/5, they were 0.49 (±2.50°), 0.40 (±5.00°) and 0.06 (±7.50°).

Table 1 shows the mean LCVA values and standard deviations (SD) for the CSC and B_175/5 designs under various rotational conditions (upper part of the table), with data averaged across positive and negative rotations. Statistically significant differences in mean LCVA between CSC and TMT were found only at a 0.00° rotation. No significant differences were observed in the other positions (all *p* > 0.05). In the CSC design, mean LCVA decreased from +0.01 logMAR (at 0.00°) to +0.20 logMAR (at 7.50°). The B_175/5 design showed a smaller decline, from +0.11 to +0.18 logMAR.

**Table 1:** The upper section presents mean LCVA ± SD values by design and rotation.

		CSC	B_175/5
LCVA mean ± SD (logMAR)	On-axis	+0.01 ± 0.08	+0.11 ± 0.06
	│2.50°│	+0.06 ± 0.06	+0.11 ± 0.05
	│5.00°│	+0.13 ± 0.05	+0.12 ± 0.06
	│7.50°│	+0.20 ± 0.08	+0.18 ± 0.07
LCVA dif. (logMAR) from on-axis and *p*-value	│2.50°│	+0.05; *p* = 0.02^a^	+0.01; *p* = 0.91
	│5.00°│	+0.12; *p* < 0.001^a^	+0.01; *p* = 0.27
	│7.50°│	+0.19; *p* < 0.001^a^	+0.07; *p* < 0.001^a^

The lower section displays LCVA differences relative to the on-axis position. Positive (counterclockwise) and negative (clockwise) rotations were averaged for each condition. *B_175/5* Optimal *TMT* (trizonal multi-toric) design with cylinder axes at 175° (first ring) and 5° (second), and area distribution of [40, 30, 30]%.

*CSC* conventional sphero-cylindrical, *LCVA* low-contrast visual acuity.

^a^Statistically significant differences (*p* < 0.05).

The lower part of Table 1 summarises the experimental LCVA differences between each rotated condition and the on-axis position. In the CSC design, all rotated conditions showed statistically significant reductions compared with the aligned position, with a loss of nearly 2 lines at a 7.50° rotation (a loss of +0.19 logMAR). For the B_175/5 design, a significant difference was observed only at 7.50°, corresponding to a decrease of nearly 1 line (a loss of +0.07 logMAR), indicating greater rotational stability.

**Fig. 4:**
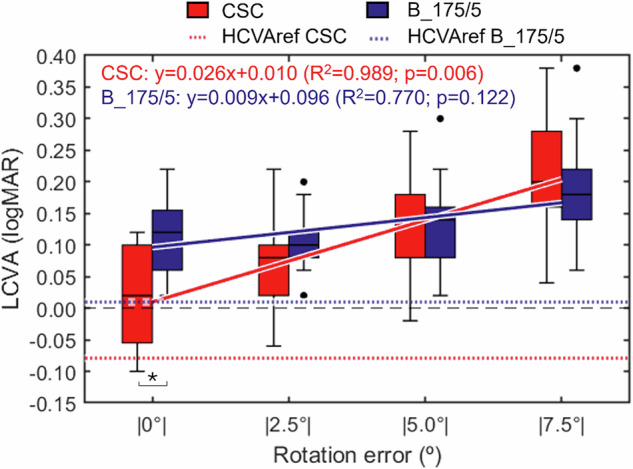
Comparison of LCVA between CSC (red) and B_175/5 (blue) designs under different rotational errors. The *x*-axis represents the magnitude of the rotational error (on-axis, │2.50°│, │5.00°│, │7.50°│). High-contrast visual acuity at the aligned position (HCVAref) is shown as discontinuous horizontal lines, following the same colour scheme as each design. Continuous lines represent the linear fitting for each design, including the corresponding coefficients of determination (*R*²) and *p*-values. *Statistically significant differences (*p* < 0.05). CSC conventional sphero-cylindrical, B_175/5 Optimal TMT (trizonal multi-toric) design with cylinder axes at 175° (first ring) and 5° (second), and area distribution of [40, 30, 30]%. CSC conventional sphero-cylindrical, HCVA high-contrast visual acuity, LCVA low-contrast visual acuity.

Figure 4 illustrates the performance of the CSC (red) and B_175/5 (blue) designs under different degrees of rotational misalignment, showing the variation in logMAR LCVA across rotation angles. The distance between the HCVAref and LCVA values represents the loss of VA; thus, a greater distance indicates a larger decrease in VA compared with high-contrast performance. At the aligned position, both designs exhibited a comparable reduction in VA when transitioning from high to low contrast, with a difference between the mean LCVA and HCVAref values of approximately +0.10 logMAR. However, as the rotational error increased, a clear difference in the behaviour of the two designs became evident. The CSC design showed a progressive decrease in LCVA; at ±7.50° rotation, the difference between HCVAref and the mean LCVA reached approximately +0.27 logMAR, reflecting a substantial loss of visual quality. In contrast, the B_175/5 design also showed a reduction in LCVA, although of smaller magnitude under the same conditions; at ±7.50°, the difference was about +0.17 logMAR, significantly lower than that of the CSC, indicating greater robustness to rotational error under low-contrast conditions.

For completeness, a linear fit was applied to the mean LCVA values to illustrate the overall trend as a function of rotational misalignment. The CSC design exhibited a significantly steeper slope (*y* = 0.026*x* + 0.010, *R*² = 0.99, *p* = 0.006), indicating a more pronounced decline in LCVA with increasing rotation. In contrast, the B_175/5 design showed a gentler slope (*y* = 0.009*x* + 0.096, *R*² = 0.77, *p* = 0.12), suggesting that the loss of visual acuity induced by rotation is considerably smaller.

## Discussion

It is estimated that nearly one-third of potential CL wearers require an astigmatic correction [24]. Among them, approximately 15% present with corneal oblique astigmatism, which poses greater challenges for achieving stable rotation with toric soft CL [25, 26]. Eyelid tension, palpebral aperture size and several anatomical lid angles have also been identified as important factors influencing rotational stability [27, 28]. Achieving stable rotation remains one of the most significant clinical challenges when correcting moderate-to-high astigmatism with toric soft CL.

The exact prevalence of clinically significant rotational misalignment, which may result in suboptimal or unstable visual performance in the absence of lens replacement or axis realignment, has not been documented consistently in the literature. However, according to the ‘3.33% rule’, a 10.00° rotation of a 3.50 D cylindrical correction (corresponding to approximately 3% of the astigmatic population [29]) is estimated to reduce its corrective efficacy by about one-third, inducing a spherical equivalent error of approximately 0.60 D (0.30 D for a 5.00° rotation). In comparison, a 1.75 D cylindrical correction, observed in roughly 13% of individuals [29], exhibits a smaller degradation under the same conditions, producing a spherical equivalent error of about 0.30 D.

In practice, while most toric lens wearers achieve satisfactory correction [30, 31], a notable minority may experience suboptimal vision due to lens instability, necessitating reassessment of lens design or brand to ensure a stable and functional fit. The TMT design proposed in this work helps achieve a functional fit by minimising the degradation of image quality caused by rotational errors, making vision less sensitive to lens rotation and potentially reducing chair time for the practitioner.

On the other hand, improving the tolerance of image quality from rotational misalignment of lenses to compensate for astigmatism could substantially reduce the number of stock-keeping units (SKUs) required. Higher tolerance allows each lens axis to maintain perceptually acceptable visual performance over a wider range of orientations. For instance, assuming 25 spherical powers (−8.00 to +3.00 D in 0.25 D steps) and one cylindrical power of −3.50 D, a ±5.00° tolerance requires 18 axis positions (810 SKUs), whereas increasing the tolerance to ±7.50° reduces this to 12 axes (540 SKUs), representing a 33% stock reduction. This example is simplified; for a detailed analysis of factors influencing lens inventories, see Young et al. [24].

This study used a two-phase approach to evaluate the potential performance of TMT designs. In the first phase, simulated retinal images were utilised to analyse the performance of various TMT configurations under rotational errors up to ±7.50° with 2.50° steps, enabling identification of an optimal TMT design. The second phase focused on evaluating the performance of this optimised design under low-contrast conditions using an adaptive visual simulator in a real patient sample. In close relation with multifocal lenses, the goal was to determine whether TMT elements induce a significant reduction in VA in low-contrast scenarios due to its multi-toric nature.

In the first phase, the image simulations with CSC correction (top row of Fig. 2) illustrate how rotational misalignment produces a blur corresponding to the PSF at the circle of least confusion, with minimal lateral asymmetry. This behaviour is consistent with a case of mixed astigmatism with zero equivalent sphere. Moreover, increasing rotational misalignment leads to higher residual astigmatism and consequently broadens both the PSF and the perceived blur. For the present case of a 3.50 D astigmatic correction, 2.50° rotation results in a residual astigmatism of 0.30 D (equivalent to 0.15 D sphere). This agrees with the simulated image (Fig. 2), where this blur level still allows optotype legibility. Conversely, the loss of legibility of the +0.00 logMAR optotype at 5.00° of rotation is consistent with an equivalent spherical error of 0.30 D. The image simulations corresponding to the TMT designs (second-to-last row in the same figure) demonstrate a different behaviour, showing an extended tolerance to rotational errors.

A comparative evaluation of the impact of central zone size (with SC_0 power) on visual performance revealed that a 20% central zone (Design A_175/5) resulted in suboptimal visual quality at the aligned position (0.00°). In contrast, when the central zone comprised 40% (Designs B_175/5, D_175/5), visual quality was acceptable without rotations and maintained stability against rotational errors up to ±7.50°. While a 60% central zone (Design C_175/5) provided maximum visual quality at 0.00°, it exhibited reduced tolerance to rotation. Consequently, a 40% central zone is considered optimal for balancing aligned visual performance and rotational stability.

Beyond the proportion of the central area, the symmetry of the peripheral rings also plays a crucial role in the performance of TMT designs. The 175/5 configurations (e.g., Design B_175/5) and 5/175 configurations (e.g., Design B_5/175) were evaluated, which differ in the rotational orientation of the cylindrical axes in the peripheral rings. The results showed that the designs’ behaviour was not symmetric with respect to positive and negative rotations: B_175/5 performed better with negative rotations, while its mirrored counterpart, B_5/175 design, exhibited greater tolerance to positive rotations.

This asymmetry is attributable to the greater influence of the first ring relative to the second, suggesting that rings closer to the optical centre have a more pronounced impact on visual performance. This finding has meaningful implications for TMT design optimisation, as configurations could be customised based on the eye’s dominant rotation direction, which commonly trends nasally in contact lens wearers [32].

In the experimental phase of the study, results corroborated the simulation findings regarding tolerance behaviour to different rotation errors. At 0.00°, the CSC design outperformed the B_175/5 design; however, this advantage diminished with ±2.50° rotations and slightly reversed at ±7.50°, where the B_175/5 design exhibited better mean LCVA values, though without statistically significant differences. This trend is consistent with findings from a previous study using HCVA [9].

Although both designs experienced a reduction in LCVA with increasing rotation, the deterioration rate was more pronounced for the CSC design, with losses reaching +0.19 logMAR (≈2 lines) at ±7.50°. In contrast, the B_175/5 design demonstrated greater rotational tolerance with a smaller loss of +0.07 logMAR.

When comparing LCVA with HCVAref in the aligned position, the B_175/5 design showed a reduction of approximately −0.10 logMAR. This result aligns with literature reports on analogous optical systems (such as multifocal lenses) where the presence of zones with different optical powers led to reduced contrast sensitivity [33, 34]. Interestingly, the CSC design, despite its conventional structure, exhibited a comparable loss (in relative terms) under the same conditions.

Although baseline VA with TMT lenses was lower than with standard toric corrections, the greater stability of VA across different rotational conditions may represent a clinically relevant advantage. In real-life use, patients often prioritise stable and ‘comfortable’ vision over the absolute maximum VA measured under ideal conditions. Such a balance between peak VA and tolerance to rotation resembles the trade-off commonly accepted with multifocal lenses for presbyopia, where simultaneous vision reduces image quality but is nevertheless widely adopted because it supports functional performance in diverse tasks. Whether a similar level of acceptance will apply to TMT designs remains to be determined. Moreover, the relatively preserved VA under low-contrast conditions (+0.10 logMAR) suggests that TMT performance may still be adequate for daily demands, while its stability could facilitate perceptual adaptation to residual blur [35].

Nevertheless, as a pioneering study in the field of TMT designs, certain limitations should be acknowledged. Evaluations were performed using a single cylindrical power, which limits generalisability since rotational tolerance varies with cylinder magnitude [5]. However, taking into account that typical rotational errors achievable with current toric stabilisation systems range from 2.25° to 8.75° [36], the 3.50 D cylinder used in this work seems to be a good example to demonstrate the potential advantages of TMT designs because, according to the ‘3.33% rule’, a 7.50° rotational misalignment induces ≈0.45 D of spherical equivalent error in conventional toric CL. Following this reasoning, lower cylindrical powers may also benefit from TMT designs, particularly in cases where stabilisation systems fail to maintain rotational alignment to a higher degree.

From a realistic perspective, it is important to highlight that pupil size dynamics and lens decentration were not considered in this work. Ignoring decentration may lead to an overestimation of visual quality. Conversely, the experimental procedure does not allow patients to adapt to the type of image produced by the CSC and TMT designs. As a result, the lack of adaptation may lead to an underestimation of the visual quality achievable with elements exhibiting more unusual optical properties, such as TMT designs.

Therefore, future research should focus on the manufacture of the optimal TMT design for clinical evaluation under real-use conditions, including an adaptation period to capture long-term user perception and clinical effectiveness better. Pre-registered and double-blind protocols should then be implemented to measure HCVA, LCVA, contrast sensitivity and lens stability (rotation and decentration) objectively. Additionally, subjective outcomes will be assessed using structured patient questionnaires that evaluate visual dysphotopsias (e.g., halos), comfort and overall satisfaction [37].

## Conclusion

For a 40% central zone size (correctly aligned), the TMT designs provided optimal, balancing aligned vision with rotational stability. TMT designs with balanced peripheral ring proportions also contributed to this robust performance.

Asymmetry in TMT design performance was observed with positive and negative rotations, with a greater influence from the ring closer to the centre. This suggests a potential for design customisation based on the eye’s typical rotation sense.

In the aligned position, the B_175/5 designs resulted in a reduction in LCVA (approximately −0.10 logMAR compared with HCVA). Notably, the conventional CSC design exhibited a comparable loss under this same condition.

Under rotational errors, TMT designs significantly outperformed CSC lenses in maintaining LCVA. While both designs experienced some LCVA reduction with rotation, the deterioration was less pronounced with TMT designs.

## Data Availability

No datasets were generated or analysed during the current study.
